# *PTPN22* intron polymorphism rs1310182 (c.2054-852T>C) is associated with type 1 diabetes mellitus in patients of Armenian descent

**DOI:** 10.1371/journal.pone.0286743

**Published:** 2023-06-14

**Authors:** Robert Žak, Lusine Navasardyan, Ján Hunák, Jiřina Martinů, Petr Heneberg

**Affiliations:** 1 Third Faculty of Medicine, Charles University, Prague, Czech Republic; 2 Department of Endocrinology, Yerevan State Medical University after Mkhitar Heratsi, Yerevan, Armenia; Unicamillus, Saint Camillus International University of Health Sciences, ITALY

## Abstract

Protein tyrosine phosphatase, nonreceptor type 22 (*PTPN22*), is an archetypal non-HLA autoimmunity gene. It is one of the most prominent genetic contributors to type 1 diabetes mellitus outside the HLA region, and prevalence of its risk variants is subject to enormous geographic variability. Here, we address the genetic background of patients with type 1 diabetes mellitus of Armenian descent. Armenia has a population that has been genetically isolated for 3000 years. We hypothesized that two *PTPN22* polymorphisms, rs2476601 and rs1310182, are associated with type 1 diabetes mellitus in persons of Armenian descent. In this association study, we genotyped the allelic frequencies of two risk-associated *PTPN22* variants in 96 patients with type 1 diabetes mellitus and 100 controls of Armenian descent. We subsequently examined the associations of *PTPN22* variants with the manifestation of type 1 diabetes mellitus and its clinical characteristics. We found that the rs2476601 minor allele (c.1858T) frequency in the control population was very low (q = 0.015), and the trend toward increased frequency of c.1858CT heterozygotes among patients with type 1 diabetes mellitus was not significant (OR 3.34, 95% CI 0.88–12.75; χ^2^ test *p* > 0.05). The control population had a high frequency of the minor allele of rs1310182 (q = 0.375). The frequency of c.2054-852TC heterozygotes was significantly higher among the patients with type 1 diabetes mellitus (OR 2.39, 95% CI 1.35–4.24; χ^2^ test *p* < 0.001), as was the frequency of the T allele (OR 4.82, 95% CI 2.38–9.76; χ^2^ test *p* < 0.001). The rs2476601 c.1858CT genotype and the T allele correlated negatively with the insulin dose needed three to six months after diagnosis. The rs1310182 c.2054-852CC genotype was positively associated with higher HbA_1c_ at diagnosis and 12 months after diagnosis. We have provided the first information on diabetes-associated polymorphisms in *PTPN22* in a genetically isolated Armenian population. We found only a limited contribution of the prototypic gain-of-function *PTPN22* polymorphism rs2476601. In contrast, we found an unexpectedly close association of type 1 diabetes mellitus with rs1310182.

## Introduction

Armenia has a population that has been genetically isolated since the end of the Bronze Age, 3000 years ago. Armenians were found to have a genetic affinity to (1) Spaniards, Italians, and Romanians from Europe; (2) Lebanese, Jews, Druze, and Cypriots from the Near East; and (3) Georgians and Abkhazians from the Caucasus [1]. Type 1 diabetes mellitus is common in Armenia; the prevalence of type 1 diabetes mellitus in Armenia is 0.048% among persons 0–14 years old and 0.063% among persons 0–19 years old [2]. These values are one order of magnitude below those experienced in Sardinia or Finland, and generally lower compared to most European countries or the United States, but higher compared to South-East and North-East Asia. The genetic background of Armenian patients with type 1 diabetes mellitus is poorly understood.

Outside the HLA region, one of the most prominent genetic contributors to autoimmune diseases, including type 1 diabetes mellitus, is protein tyrosine phosphatase, nonreceptor type 22 (*PTPN22*), which encodes lymphoid tyrosine phosphatase (LYP). *PTPN22* is considered an archetypal non-human leukocyte antigen (HLA) autoimmunity gene [3]. It is expressed mainly in hematopoietic cells and may affect autoimmunity. LYP binds the Src homology 3 (SH3) domain of the C-terminal Src kinase (CSK) and is involved in the downregulation of T cell receptor and B cell receptor signaling when dissociated from CSK [4]. In mice, it regulates the numbers of regulatory T cells (T_reg_ cells) and follicular T helper cells [5, 6].

*PTPN22* is known for its functional polymorphism rs2476601 (c.1858C>T, p.620R>W). This missense point variation causes a gain-of-function variant of LYP. This mutation of the P1 linkage domain decreases its affinity to CSK threefold and thus leads to increased CSK activity [7]. The variant leads to increases in the frequencies of total and naïve CD4^+^CD25^+^CD127^low^FOXP3^+^ T_reg_ cells (CD = cluster of differentiation; FOXP3 = forkhead box P3) [8]. This gain-of-function variant of LYP is associated with many autoimmune diseases, including type 1 diabetes mellitus, rheumatoid arthritis, systemic lupus erythematosus, vitiligo, and others [9]. The prevalence of rs2476601 risk allele is subject to enormous geographic variability, with the highest frequencies in Northern Europe [10]. It is rare in Sub-Saharan Africa and South Asia and nearly absent in East Asia. Small interfering RNA (siRNA)-based therapies that target the rs2476601 variant are currently under development [11, 12]. These novel immunotherapeutic approaches targeting the rs2476601 variant represent personalized treatments that can be applied to patients harboring this variant in populations of European descent.

The second polymorphism, we examined in the present study, rs1310182 (c.2054-852T>C), is much less understood. It is a point mutation in an intronic region that carries a putative transcription factor-binding site, as described by Carlton et al. [13]. They found that this polymorphism was associated with rheumatoid arthritis independent of the rs2476601 status of the examined patients of European descent. Interestingly, later reports often found a close association of rs1310182 with various autoimmune diseases but disagreed on which allele is associated with the disease. The risk allele was C in Japanese patients with type 1 diabetes mellitus [14], Han Chinese patients with allergic rhinitis [15], Norwegian patients with rheumatoid arthritis [16], and Iranian patients with juvenile idiopathic arthritis [17], systemic lupus erythematosus [18] (in this study, the table with results refers to C as the disease-associated allele, whereas the text refers to T as the disease-associated variant), and ulcerative colitis [19]. The risk allele was T in patients of European descent with rheumatoid arthritis [13] or late-onset autoimmune diabetes of adults (LADA) [20] and patients of Iranian descent with celiac disease [21] and chronic spontaneous urticaria [22]. There was no association reported in studies of patients with type 1 diabetes mellitus from Sardinia [23] and Iran [24], various monogenic types of diabetes mellitus from the Czech Republic [25], rheumatoid arthritis from China [26], psoriasis from the United Kingdom [27], chronic spontaneous urticaria from Poland [28], Behcet’s disease from China [29], Vogt–Koyanagi–Harada syndrome and ankylosing spondylitis from China [30], and autoimmune thyroid disease from Japan [31]. The rejection risk of liver transplants is also independent of rs1310182 [32, 33]. In Czech patients with LADA, the CC genotype was more common in women and was associated with higher hemoglobin A_1c_ (HbA_1c_) [20]. The geographic variability in the frequency of this allele in random populations is negligible.

In the present study, we tested the hypothesis that rs2476601 and rs1310182 are associated with type 1 diabetes mellitus in persons of Armenian descent. We tested the frequencies of these polymorphisms both in the general population and in children with type 1 diabetes mellitus. We correlated the presence of minor alleles and genotypes with the clinical characteristics of the examined patients.

## Materials and methods

### Selection criteria

From September 2017 through June 2019, we recruited two groups of persons of Armenian descent for genetic testing of *PTPN22* polymorphisms. We applied the following absolute inclusion criteria:

1. Type 1 diabetes mellitus (n = 96, 40 M, 56 F):
  - Fasting C-peptide <200 pmol/L, and
  - HbA_1c_ 6.5% or higher
  - Age at diagnosis <25 years
2. Nondiabetic controls (n = 100 of undisclosed sex):
  - Fasting C-peptide >260 pmol/L, and
  - HbA_1c_ <5.7%

The patients fulfilled the type 1 diabetes mellitus diagnostic criteria according to the most recent version of the ADA guidelines [34]. Exclusion criteria were: suspected non-type 1 diabetes mellitus (type 2 diabetes, maturity-onset diabetes of the young, secondary diabetes, etc.), declined enrollment in the study by the patients or their parents, and initial treatment for diabetes outside of the study department for more than five days. Thyroiditis was used as an exclusion criterion for the nondiabetic controls.

The clinical features of the included patients with type 1 diabetes mellitus are described in Table 1. Both the patients and the controls were of Armenian descent. All were referred for clinical reasons to the Endocrinology Clinic at the "Muratsan" University Hospital Complex, Yerevan State Medical University after Mkhitar Heratsi. We obtained written informed consent from each subject.

**Table 1:** Clinical features of the patients with type 1 diabetes mellitus. Data are shown as the mean±SD (range) unless stated otherwise.

Gender Variable	M (N = 40)	F (N = 56)
Age at diagnosis [years]	8.6±5.8 (0.4–24)	9.4±4.7 (1.0–22)
C-peptide [ng mL^-1^]	0.5±0.4 (0.1–2.0)	0.5±0.3 (0.2–1.9)
HbA_1c_ at diagnosis [NGSP %]	9.8±2.1 (6.6–15.5)	10.0±1.9 (6.7–14.3)
HbA_1c_ six months after diagnosis [NGSP %]	7.0±1.0 (4.9–9.6)	7.2±1.1 (4.9–10.1)
HbA_1c_ 12 months after diagnosis [NGSP %]	7.7±1.2 (4.9–9.2)	8.3±1.9 (5.5–13.1)
TSH	3.1±1.5 (0.8–8.0)	2.8±1.5 (0.0–7.9)
Autoantibodies	No. of patients positive / tested (%)	No. of patients positive / tested (%)
Anti-GAD ≥5.0 IU * mL^–1^	3 / 15 (20%)	8 / 12 (67%)
Anti-Insulin ≥5.0 IU * mL^–1^	3 / 13 (23%)	1 / 11 (9%)
Anti-IA-2 ≥5.0 IU * mL^–1^	10 / 15 (67%)	3 / 12 (25%)
Urine ketons	No. of patients (%):	No. of patients:
3+	25 (63%)	42 (76%)
2+	6 (15%)	4 (7%)
1+	5 (13%)	2 (4%)
0	4 (10%)	7 (13%)
Acidosis	No. of patients (%):	No. of patients (%):
Present	14 (36%)	22 (41%)
Absent	25 (64%)	32 (59%)
Liver ultrasonography	No. of patients (%):	No. of patients (%):
Increased signal	4 (10%)	8 (15%)
Normal signal	36 (90%)	44 (85%)
Insulin dose three to six months after diagnosis	0.4±0.3 (0.0–1.2)	0.5±0.3 (0.0–1.0)

### Clinical and laboratory characterization

At the time of diagnosis, we measured fasting C-peptide and HbA_1c_, anti-glutamic acid decarboxylase (GAD), anti-insulin and anti-islet antigen 2 (IA-2) autoantibodies, thyroid-stimulating hormone (TSH), and urine ketones. We also recorded the presence of acidosis episodes and the need for insulin treatment three to six months after the diagnosis and performed abdominal ultrasonography. We repeated the HbA_1c_ measurements six and 12 months after the diagnosis. We determined HbA_1c_ by high-performance liquid chromatography using the Diabetes Control and Complications Trial (DCCT) % units [35]. We measured the fasting serum C-peptide and TSH levels by chemiluminescent immunoassay method using Cobas E 411 analyzer (Roche diagnostics, Basel, Switzerland). We detected the autoantibodies using radioimmunoassays from DIAsource ImmunoAssays S.A. (Louvain-la-neuve, Belgium). Furthermore, we measured ketone bodies using Combur-Test strip with the Urisys 1100 semi-automated urine testing analyzer (Roche Diagnostics, Basel, Switzerland).

### Blood collection

After obtaining informed consent, we collected blood samples for DNA analyses by venipuncture in EDTA-containing tubes and processed the blood as described below. All of the measurements and collections were performed by us or by trained nurses and technicians of the study institutions.

### Genotyping

We purified peripheral blood leukocytes using Ficoll, washed them using physiological saline solution and froze the pellets at or below -75°C until further processing. We then isolated DNA from the peripheral blood leukocytes using the DNeasy Blood & Tissue Kit (Qiagen, Hilden, Germany) and ensured that the isolated DNA was of acceptable purity (A_260_/A_280_ ratio of 1.7–2.0) and concentration (≥30 ng μL^-1^). Next, we genotyped the following *PTPN22* SNPs that have previously been proposed to be associated with autoimmune diseases: rs2476601 (c.1858C>T) and rs1310182 (NM_015967.7:c.2054-852T>C).

We tested the polymorphism rs2476601 by Sanger sequencing (133 samples) or by using a polymerase chain reaction–restriction fragment length polymorphism (PCR-RFLP) (the remaining samples; with five randomly chosen samples validated by Sanger sequencing). To amplify the rs2476601 locus, we used a nested PCR design with the following primers: 1^st^ pair, fw: CCTCCTGGGTTTGTACCTTAAGAG, rv: CTGGAATTAAAGGCATGAGCCACCATG; 2^nd^ pair, fw: TCACCAGCTTCCTCAACCACA, rv: GATAATGTTGCTTCAACGGAATTT. We designed the 1^st^ pair of primers for the purpose of this study (using Primer3 [36]) and used the 2^nd^ pair of primers designed by Bottini et al. [37]. We amplified the extracted DNA using the following PCR mix: 13.25 μL of PCR water, 5 μL of 5× concentrated Q5 buffer, 0.5 μL of dNTPs (10 mM), 2.5 μL of 5′ primer (10 μM), 2.5 μL of 3′ primer (10 μM), 0.25 μL of Q5 DNA polymerase (2 U μL^-1^) (Thermo Fisher Scientific, Waltham, MA), and 1 μL of extracted genomic DNA (30–60 ng μL^-1^). The total volume was 25 μl. PCR was carried out for 35 cycles with 10 s denaturation at 98°C, 10 sec annealing at 55°C, followed by 2 min extension at 72°C. Cycling was started by 30 sec denaturation at 98°C and terminated by 2 min incubation at 72°C. The experiments were performed using an Eppendorf Mastercycler (Eppendorf, Hamburg, Germany). We purified the PCR products using a NucleoSpin Gel and PCR Clean-up kit (Macherey-Nagel, Allentown, PA) and subjected them either to Sanger sequencing using an ABI 3130 DNA Analyzer (Applied Biosystems, Foster City, CA) or to an overnight restriction with XcmI. We visualized the sequences using FinchTV v 1.4.0 (Geospiza, Seattle, WA) and processed them in MEGA5.2 [38]. In each run of the restriction analyses, we included a DNA sample as a positive control (a known c.1858CT heterozygote). Uncleaved product length was 215 bp, while the cleaved products were 170 bp and 45 bp long.

We tested the polymorphism rs1310182 by PCR-RFLP, with four randomly chosen samples validated by Sanger sequencing. To amplify the rs1310182 locus, we used the following PCR primers: fw: AATGGACATATTTTCCCATGATGT, rv: TGCCTACTGTATGCCAGTTATTTT (designed for the purpose of this study using Primer3 [36]). The PCR conditions were identical to those for rs2476601. We then purified the PCR products using a NucleoSpin Gel and PCR Clean-up kit and subjected them to overnight restriction with PmlI. We retested all individuals revealed to be heterozygous in the first round of PCR-RFLP to exclude incomplete cleavage. In each run of the restriction analyses, we included a DNA sample as a positive control (known c.2054-852TC heterozygote). Uncleaved product length was 285 bp, while the cleaved products were 140 bp and 145 bp long. Examples of agarose blots with RFLP results and Sanger sequencing results are disclosed in S1 Fig for both rs2476601 and rs1310182.

### Statistical analyses

We calculated the desired sample size by estimating the frequency of the rs1310182 minor allele to be 35% in the control population and 55% in patients with type 1 diabetes mellitus. Considering dichotomous endpoints and two independent groups of patients, the tested groups should include 96 patients or controls to reach 80% power at α = 0.05. The power analysis was performed with ClinCalc (ClinCalc LLC., Arlington Heights, IL).

We present the clinical characteristics of the patients with type 1 diabetes mellitus as the mean±SD (range) or absolute counts (%). We further described the differences by calculating odds ratios (ORs) and corresponding 95% confidence intervals (CIs). We used the χ^2^ test to analyze the significance of the calculated OR. We used PERMANOVA to analyze the differences in the genotype and allelic frequencies between the patients with type 1 diabetes mellitus and the controls. The rs2476601 c.1858C>T variant functions in an autosomal dominant fashion with increased clinical penetrance in carriers who are homozygous [39], while the genetic model for rs1310182 is unknown. We used principal component analysis (PCA) and Pearson product-moment correlation analysis to check for the contribution of individual genotypes and alleles. We calculated the allelic frequencies and analyzed the consistency of the distribution of genotypes with Hardy-Weinberg’s law. The calculations were performed in PAST v 2.14, SigmaPlot v 12.0 and Gene-calc.

### Statement of ethics

This study was approved by the Ethics Committees of the Third Faculty of Medicine at Charles University and Yerevan State Medical University after Mkhitar Heratsi. The decisions are not provided with numbers. All methods were performed in accordance with the relevant guidelines and regulations. Written informed consent was obtained from the participants (or their parents).

## Results

### *PTPN22* signature of Armenian patients with type 1 diabetes mellitus

The concordance rates between the PCR-RFLP and Sanger sequencing results were 100% for both tested SNPs. Both SNPs were variable in Armenian patients with type 1 diabetes mellitus and controls. PERMANOVA of the two analyzed polymorphisms, exploring the genotype and allelic frequencies, revealed significant differences between the patients with type 1 diabetes mellitus and the controls (permutation n = 9999; F = 4.587; *p* = 0.01). Subsequent PCA revealed type 1 diabetes mellitus to be negatively associated with the rs1310182 c.2054-852CC genotype and positively associated with the c.2054-852T allele. In contrast, the rs2476601 genotypes and alleles contributed negligibly (S2 Fig).

Concerning rs2476601, the control population had a low frequency of this polymorphism; there were only three c.1858CT heterozygotes among the 100 analyzed controls (q = 0.015; equal to the minor allele frequency). However, there were nine c.1858CT heterozygotes among the 96 analyzed patients with type 1 diabetes mellitus (q = 0.047; equal to the minor allele frequency). The c.1858TT homozygotes were absent from both study populations. The trend toward a higher representation of c.1858CT heterozygotes among the patients with type 1 diabetes mellitus was not significant when compared to the frequency of c.1858CT heterozygotes among the examined controls (OR 3.34, 95% CI 0.88–12.75; χ^2^ test *p* > 0.05). Both populations were in Hardy-Weinberg equilibrium (patients with type 1 diabetes mellitus: χ^2^ statistics 0.232, *p* = 0.89; controls: χ^2^ statistics 0.023, *p* = 0.99).

Concerning rs1310182, the control population had a high frequency of this polymorphism. There were 18 c.2054-852TT homozygotes and 39 c.2054-852CT heterozygotes among the 100 analyzed controls (q = 0.375). There were 25 c.2054-852TT homozygotes and 58 c.2054-852CT heterozygotes among the 96 analyzed patients with type 1 diabetes mellitus (q = 0.563). The representation of c.2054-852TT homozygotes was similar among the patients with type 1 diabetes mellitus compared to their expected frequency according to the examined controls (OR 1.60, 95% CI 0.81–3.18; χ^2^ test *p* > 0.05). In contrast, the representation of c.2054-852CT heterozygotes was significantly higher among the patients with type 1 diabetes mellitus compared to their expected frequency according to the examined controls (OR 2.39, 95% CI 1.35–4.24; χ^2^ test *p* < 0.001). The higher prevalence of the T allele among patients with type 1 diabetes mellitus was also significant (OR 4.82, 95% CI 2.38–9.76; χ^2^ test *p* < 0.001). Both populations were in Hardy-Weinberg equilibrium (patients with type 1 diabetes mellitus: χ^2^ statistics 4.969, *p* = 0.08; controls: χ^2^ statistics 2.822, *p* = 0.24). An overview of the ORs is provided in Table 2.

**Table 2:** *PTPN22* signature of Armenian patients with type 1 diabetes mellitus and nondiabetic controls. Data are shown as the number of patients/controls with the respective genotype, OR ± 95% CI, with the associated χ^2^ test *p* values.

Polymorphism, genotype / allele	N of patients/controls (%)	OR (95% CI)	χ^2^ test *p* value
rs2476601			
c.1858CC	87 (90.6%) / 97 (97.0%)		
c.1858CT	9 (9.4%) / 3 (3.0%)	3.34 (0.88–12.75)	> 0.05
c.1858TT	0 (0%) / 0 (0%)	N/A	N/A
c.1858C	183 (95.3%) / 197 (98.5%)		
c.1858T	9 (4.7%) / 3 (1.5%)	3.23 (0.86–12.11)	> 0.05
rs1310182			
c.2054-852CC	13 (14.0%) / 43 (43.0%)		
c.2054-852CT	58 (60.4%) / 39 (39.0%)	2.39 (1.35–4.24)	< 0.001
c.2054-852TT	25 (26.0%) / 18 (18.0%)	1.60 (0.81–3.18)	> 0.05
c.2054-852C	84 (43.8%) / 125 (62.5%)		
c.2054-852T	108 (56.2%) / 75 (37.5%)	4.82 (2.38–9.76)	< 0.001

### Correlations of *PTPN22* genotypes with clinical features of patients with type 1 diabetes mellitus

The correlation analysis revealed that the rs2476601 c.1858CT genotype and the presence of the T allele correlated negatively with the male sex among the patients (Pearson r = -0.199, *p* = 0.05 for both), while the c.1858CC genotype was correlated positively with the male sex among the patients (Pearson r = 0.199, *p* = 0.05). The rs2476601 c.1858CT genotype and the presence of the T allele also correlated negatively with the insulin dose needed three to six months after diagnosis (Pearson r = -0.265, *p* = 0.04 for both). In contrast, the c.1858CC genotype was correlated positively with the insulin dose needed three to six months after diagnosis (Pearson r = 0.265, *p* = 0.04) (S3 Fig). All the correlations were relatively weak.

The rs1310182 c.2054-852CC genotype was positively associated with a higher HbA_1c_ at diagnosis (Pearson r = 0.240, *p* = 0.02) and 12 months after diagnosis (Pearson r = 0.293, *p* = 0.04) (S3 Fig).

Other parameters, including all three tested diabetes-associated autoantibodies, were independent of the presence of the tested genotypes and alleles (Pearson *p* > 0.05 each) (S3 Fig). The raw data are provided in S1 File.

## Discussion

The present study provided the first evidence of the frequency of the rs2476601 c.1858CT genotype in patients with type 1 diabetes mellitus of Armenian descent and in the general Armenian population. The frequency of rs2476601 in Armenia was previously unknown. The Armenian nation has been genetically isolated. Therefore, it was unclear whether rs2476601 genotyping could be of any use analogous to its use to predict autoimmune diseases onset in European populations. It was also unclear whether a fraction of Armenian patients with type 1 diabetes mellitus could be sensitive to the newly developed rs2476601 c.1858T-targeting therapies [11, 12].

Unexpectedly, we identified rs1310182 as a polymorphism closely associated with type 1 diabetes mellitus in the Armenian population. Rs1310182 is an intronic polymorphism located in a putative transcription factor-binding site of the *PTPN22* gene, but this site also overlaps with the adaptor-related protein complex 4 subunit beta 1 (*AP4B1*) *antisense RNA 1* gene. Previous reports on its association with autoimmune diseases were conflicting [13, 14, 16, 20, 23, 26, 28]. Some of the previous studies reported no significant association; other reported the association of some autoimmune diseases with the C allele, and only a fraction of previous studies reported the association of a similar spectrum of autoimmune diseases with the T allele of the same polymorphism. Here, we found a close association of rs1310182 c.2054-852CT heterozygotes and the T allele with type 1 diabetes mellitus.

We noticed a higher frequency of the rs2476601 c.1858CT genotype and the T allele in male patients with type 1 diabetes mellitus. The sex-specific association of rs2476601 with various autoimmune diseases is controversial. Previously, Kahles et al. [40] reported a close association between 1858T and type 1 diabetes mellitus in females but no association in males. Later, Cinek et al. [41] and Heneberg et al. [20] questioned the gender bias of the pro-diabetic effects of the 1858T allele. These inconsistencies may be a result of the relatively small sample sizes.

We found that the analyzed polymorphisms were associated with the insulin dose needed three to six months after diagnosis, HbA_1c_ at diagnosis, and HbA_1c_ 12 months after diagnosis. The association of polymorphic rs2476601 with higher levels of C-peptide at diagnosis and lower insulin requirements six months after diagnosis has already been reported [42]. Additionally, the positive association of the rs1310182 c.2054-852CC genotype with higher HbA_1c_ was previously reported but only in patients with LADA and measured at various postdiagnosis times [20]. Interestingly, wild-type *PTPN22* (rs2476601 c.1858CC) combined with *FCRL3* rs7528684 NG_023241.1:g.4832CC was previously associated with higher HbA_1c_ at diagnosis but lower HbA_1c_ at the sixth and later months in patients with type 1 diabetes mellitus [43].

Limitations of the present study consist of enrolling a sample size that would be sufficient to uncover associations of strength similar to those of rs2476601 in Western- or Central-European populations. However, given that we found that the frequency of the rs2476601 minor allele is low among control individuals from the study population, a larger sampling effort would be necessary to confirm the association of rs2476601 with type 1 diabetes mellitus in the study region. In line with this finding, we found a trend toward higher representation of the rs2476601 minor allele among patients with type 1 diabetes mellitus in the study population, but due to the low number of heterozygotes in both controls and patients with type 1 diabetes mellitus, the association was not significant. These results show that only a small fraction of Armenian patients with type 1 diabetes mellitus would benefit from recently developed siRNA-based therapies that target the rs2476601 variant [11, 12], which is in contrast to the populations with type 1 diabetes mellitus in Western and Central Europe. Another limitation of the study stems from limited knowledge of the health status of the controls. Although we selected the controls to be nondiabetic at the time of blood collection, we cannot exclude the possibility that they could develop type 1 diabetes in the future.

In conclusion, we provide the first information on diabetes-associated polymorphisms in *PTPN22* in a genetically isolated Armenian population. We found only a limited contribution of the prototypic gain-of-function *PTPN22* polymorphism rs2476601 c.1858C>T. In contrast, we found an unexpectedly close association of type 1 diabetes mellitus with the rs1310182 c.2054-852C>T polymorphism. The function of rs1310182 remains to be elucidated, and additional studies are needed to corroborate the present finding in patients with type 1 diabetes mellitus of other geographic origins.

## Supporting information

S1 Fig Examples of agarose blots with RFLP results and Sanger sequencing results. (A) rs2476601 and (B) rs1310182. Raw images are provided in S1 Raw images.
(PDF)

S2 Fig Association of the *PTPN22* genotypes with type 1 diabetes mellitus in patients and controls of Armenian descent. The biplot shows the outcomes of principal component analysis (PCA), which determined, whether any of the two genotyped *PTPN22* SNPs (in sum six genotypes and two minor alleles) were associated with type 1 diabetes mellitus. The PCA simply reduced the data to only two variables that represent the two most important components (Axis 1 and Axis 2) and projected the correlation of the other variables with these components. The genotypes and minor alleles are indicated by the position of the polymorphism and relevant letters; T1DM = type 1 diabetes mellitus. Axis 1 explained 46.9% of the variability (eigenvalue 0.590) and Axis 2 explained 23.1% of the variability (eigenvalue 0.291).
(TIF)

S3 Fig Volcano plot showing the Pearson product-moment correlation of clinical characteristics of the patients with type 1 diabetes mellitus with their *PTPN22* genotypes and minor alleles. The data are shown as–log_10_-transformed *p* values and corresponding Pearson correlation coefficients r. The most significant values are associated with the descriptor of the respective data point.
(TIF)

S1 File Raw data. (XLSX)

S1 Raw images Raw images. (PDF)

## References

[1] Haber M, Mezzavilla M, Xue Y, Comas D, Gasparini P, Zalloua P, et al. Genetic evidence for an origin of the Armenians from Bronze Age mixing of multiple populations. Eur J Hum Genet. 2016;24:931–936 doi: 10.1038/ejhg.2015.206 26486470 PMC4820045

[2] Boyko EJ, Karuranga S, Magliano DJ, Saeedi P, Sun H. IDF Diabetes Atlas, 10^th^ Edition. Brussels, International Diabetes Federation, 2022.

[3] Stanford SM, Bottini NB. *PTPN22*: the archetypal non-HLA autoimmunity gene. Nat Rev Rheumatol. 2014;10:602–611. 25003765 10.1038/nrrheum.2014.109 PMC4375551

[4] Vang T, Liu WH, Delacroix L, Wu S, Vasile S, Dahl R, et al. LYP inhibits T cells activation when dissociated from CSK. Nat Chem Biol. 2012;8:437–446. 22426112 10.1038/nchembio.916 PMC3329573

[5] Brownlie RJ, Miosge LA, Vassilakos D, Svensson LM, Cope A, Zamoyska R. Lack of the phosphatase *PTPN22* increases adhesion of murine regulatory T cells to improve their immunosuppressive function. Sci Signal. 2012;5:ra87. 23193160 10.1126/scisignal.2003365 PMC5836999

[6] Maine CJ, Hamilton-Williams EE, Cheung J, Stanford SM, Wicker LS, Sherman LA. *PTPN22* alters the development of regulatory T cells in the thymus. J Immunol. 2012;188:5267–5275. 22539785 10.4049/jimmunol.1200150 PMC3358490

[7] Zheng W, She JX. Genetic association between a lymphoid tyrosine phosphatase (*PTPN22*) and type 1 diabetes. Diabetes. 54;2005:906. 15734872 10.2337/diabetes.54.3.906

[8] Valta M, Gazali AM, Viisanen T, Ihantola E-L, Ekman I, Toppari J, et al. Type 1 diabetes linked *PTPN22* gene polymorphism is associated with the frequency of circulating regulatory T cells. Eur J Immunol. 2020;50:581–588. 31808541 10.1002/eji.201948378

[9] Tizaoui K, Kim SH, Jeong GH, Kronbichler A, Lee KS, Shin JI. Association of *PTPN22* 1858C/T polymorphism with autoimmune diseases: a systematic review and Bayesian approach. J Clin Med. 2019;8:347. 30871019 10.3390/jcm8030347 PMC6462981

[10] Totaro MC, Tolusso B, Napolioni V, Faustini F, Canestri S, Mannocci A, et al. *PTPN22* 1858C>T polymorphism distribution in Europe and association with rheumatoid arthritis: case-control study and meta-analysis. PLoS ONE. 2011;6:e24292. 21949702 10.1371/journal.pone.0024292 PMC3174938

[11] Pellegrino M, Ceccacci F, Petrini S, Scipioni A, De Santis S, Cappa M, et al. Exploiting novel tailored immunotherapies of type 1 diabetes: Short interfering RNA delivered by cationic liposomes enables efficient down-regulation of variant *PTPN22* gene in T lymphocytes. Nanomedicine. 2019;18:371–379. 30439564 10.1016/j.nano.2018.11.001

[12] Arena A, Belcastro E, Ceccacci F, Petrini S, Conti LA, Pagliarosi O, et al. Improvement of lipoplexes with a sialic acid mimetic to target the C1858T *PTPN22* variant for immunotherapy in endocrine autoimmunity. Front Immunol. 2022;13:838331. 35355982 10.3389/fimmu.2022.838331 PMC8959661

[13] Carlton VEH, Hu X, Chokkalingam AP, Schrodi SJ, Brandon R, Alexander HC, et al. *PTPN22* genetic variation: evidence for multiple variants associated with rheumatoid arthritis. Am J Hum Genet. 2005;77:567–581. 16175503 10.1086/468189 PMC1275606

[14] Taniyama M, Maruyama T, Tozaki T, Nakano Y, Ban Y. Association of *PTPN22* haplotypes with type 1 diabetes in the Japanese population. Hum Immunol. 2010;71:795–798. 20510318 10.1016/j.humimm.2010.05.016

[15] Ke X, Song S, Wang X, Shen Y, Kang H, Hong S. Association of single nucleotide polymorphisms of *PTPN22* and *Ctla4* genes with the risk of allergic rhinitis in a Chinese Han population. Hum Immunol. 2017;78:227–231. 27888068 10.1016/j.humimm.2016.11.008

[16] Viken MK, Olsson M, Flåm ST, Førre O, Kvien TK, Thorsby E, et al. The *PTPN22* promoter polymorphis -1123G>C association cannot be distinguished from the 1858C>T association in a Norwegian rheumatoid arthritis material. Tissue Antigens. 2007;70:190–197. 17661906 10.1111/j.1399-0039.2007.00871.x

[17] Bahrami T, Soltani S, Moazzami K, Yekaninejad MS, Salmaninejad A, Soltaninejad E, et al. Association of *PTPN22* gene polymorphisms with susceptibility to juvenile idiopathic arthritis in Iranian population. Fetal Pediatr Pathol. 2017;36:42–48. 27732119 10.1080/15513815.2016.1231249

[18] Bahrami T, Valilou SF, Sadr M, Soltani S, Salmaninejad A, Soltaninejad E, et al. *PTPN22* gene polymorphisms in pediatric systemic lupus erythematosus. Fetal Pediatr Pathol. 2020;39:13–20. 31232672 10.1080/15513815.2019.1630873

[19] Sadr M, Moazzami B, Soleimanifar N, Elhamian N, Rezaei A, Daryani NE, et al. Single nucleotide polymorphisms of *PTPN22* gene in Iranian patients with ulcerative colitis. Fetal Pediatr Pathol. 2019;38:8–13. 30636557 10.1080/15513815.2018.1543371

[20] Heneberg P, Kocková L, Čecháková M, Daňková P, Černá M. Autoimmunity-associated *PTPN22* polymorphisms in latent autoimmune diabetes of the adult differ from those of type 1 diabetes patients. Int Arch Allergy Immunol. 2018;177:57–68. 29895027 10.1159/000489225

[21] Aflatounian M, Rezaei A, Sadr M, Saghazadeh A, Elhamian N, Sadeghi H, et al. Association of *PTPN22* single nucleotide polymorphisms with celiac disease. Fetal Pediatr Pathol. 2017;36:195–202. 28481156 10.1080/15513815.2017.1290725

[22] Sadr M, Khalili N, Mohebbi B, Mosharmovahed B, Afradi P, Rezaei N. Association of *PTPN22* single nucleotide polymorphisms with chronic spontaneous urticarial. Allergol Immunopathol (Madr). 2021:49:40–45. 10.15586/aei.v49i2.53 33641292

[23] Zoledziewska M, Perra C, Orrù V, Moi L, Frongia P, Congia M, et al. Further evidence of a primary causal association of the *PTPN22* 620W variant with type 1 diabetes. Diabetes. 2008;57:229–234. 17934143 10.2337/db07-0289

[24] Abbasi F, Soltani S, Saghazadeh A, Soltaninejad E, Rezaei A, Bidoki AZ, et al. *PTPN22 s*ingle-nucleotide polymorphisms in Iranian patients with type 1 diabetes mellitus. Immunol Invest. 2017;46:409–418. 28375784 10.1080/08820139.2017.1288239

[25] Heneberg P, Malá M, Yorifuji T, Gat-Yablonski G, Lebenthal Y, Tajima T, et al. Low frequencies of autoimmunity-associated *PTPN22* polymorphisms in MODY patients, including those transiently expressing islet cell autoantibodies. Int Arch Allergy Immunol. 2015;166:189–198. 25896041 10.1159/000380853

[26] Huang J-J, Qiu Y-R, Li H-X, Sun D-H, Yang J, Yang C-L. A *PTPN22* promoter polymorphism -1123G>C is associated with RA pathogenesis in Chinese. Rheumatol Int. 2012;32:767–771. 21193990 10.1007/s00296-010-1705-x

[27] Smith RL, Warren RB, Eyre S, Ke X, Young HS, Allen M, et al. Polymorphisms in the *PTPN22* region are associated with psoriasis of early onset. Br J Dermatol. 2008;158:962–968. 18341666 10.1111/j.1365-2133.2008.08482.x PMC2342636

[28] Brzoza Z, Grzeszczak W, Rogala B, Trautsolt W, Moczulski D. *PTPN22* polymorphism presumably plays a role in the genetic background of chronic spontaneous autoreactive urticaria. Dermatology. 2012;224:340–345. 22722472 10.1159/000339332

[29] Zhang Q, Hou S, Jiang Z, Du L, Li F, Xiao X, et al. No association of *PTPN22* polymorphisms with susceptibility to ocular Behcet’s disease in two Chinese Han populations. PLoS ONE. 2012;7:e31230. 22396730 10.1371/journal.pone.0031230 PMC3292549

[30] Zhang Q, Qi J, Hou S, Du L, Yu H, Cao Q, et al. A functional variant of *PTPN22* confers risk for Vogt-Koyanagi-Harada syndrome but not for ankylosing spondylitis. PLoS ONE. 2014;9:e96943. 24816862 10.1371/journal.pone.0096943 PMC4016172

[31] Ban Y, Tozaki T, Taniyama M, Nakano Y, Ban Y, Ban Y, et al. Association of the protein tyrosine phosphatase nonreceptor 22 haplotypes with autoimmune thyroid disease in the Japanese population. Thyroid. 2010;20:893–899. doi: 10.1089/thy.2010.0104 20615141

[32] Dullin R, Koch M, Sterneck M, Nashan B, Thude H. Association between a gain-of-function variant of *PTPN22* and rejection in liver transplantation. Transplantation. 2015;99:431–437. 25073032 10.1097/TP.0000000000000313

[33] Thude H, Tiede P, Marget M, Peine S, Nashan B, Koch M. Protein tyrosine phosphatase non-receptor type 22 (*PTPN22*) gene polymorphisms in liver transplant donors and impact on acute cellular liver transplant rejection. HLA. 2020;95:40–44. 31577847 10.1111/tan.13706

[34] ElSayed NA, Aleppo G, Aroda VR, Bannuru RR, Brown FM, Bruemmer D, et al. 2. Classification and diagnosis of diabetes: Standards of care in diabetes 2023. Diabetes Care 2023;46:S19–S40. doi: 10.2337/dc23-S002 36507649 PMC9810477

[35] Maple-Brown LJ, Ye C, Retnakaran R. Area-under-the-HbA1c curve above the normal range and the prediction of microvascular outcomes: an analysis of data from the Diabetes Control and Complications Trial. Diabet Med. 2013;30:95–99. doi: 10.1111/dme.12004 22937915 PMC3843010

[36] Untergasser A, Cutcutache I, Koressaar T, Ye J, Faircloth BC, Remm M, et al. Primer3 –new capabilities and interfaces. Nucleic Acids Res. 2012;40:e115. doi: 10.1093/nar/gks596 22730293 PMC3424584

[37] Bottini N, Musumeci L, Alonso A, Rahmouni S, Nika K, Rostamkhani M, et al. A functional variant of lymphoid tyrosine phosphatase is associated with type I diabetes. Nat Genet. 2004;36:337–338. doi: 10.1038/ng1323 15004560

[38] Tamura K, Peterson D, Peterson N, Stecher G, Nei M, Kumar S. MEGA5: Molecular Evolutionary Genetics Analysis using Maximum Likelihood, Evolutionary Distance, and Maximum Parsimony Methods. Mol Biol Evol. 2011;28:2731–2739. doi: 10.1093/molbev/msr121 21546353 PMC3203626

[39] Burn GL, Svensson L, Sanchez-Blanco C, Saini M, Cope AP. Why is *PTPN22* a good candidate susceptibility gene for autoimmune disease? FEBS Lett. 2011;585:3689–3698. 21515266 10.1016/j.febslet.2011.04.032

[40] Kahles H, Ramos-Lopez E, Lange B, Zwermann O, Reincke M, Badenhoop K. Sex-specific association of *PTPN22* 1858T with type 1 diabetes but not with Hashimoto’s thyroiditis or Addison’s disease in the German population. Eur J Endocrinol. 2005;153:895–899. 16322396 10.1530/eje.1.02035

[41] Cinek O, Hradsky O, Ahmedov G, Slavcev A, Kolouskova S, Kulich M, et al. No independent role of the -1123G>C and +2740A>G variants in the association of *PTPN22* with type 1 diabetes and juvenile idiopathic arthritis in two Caucasian populations. Diabet Res Clin Pract. 2007;76:297–303. 10.1016/j.diabres.2006.09.009 17000021

[42] Blasetti A, Di Giulio C, Tumini S, Provenzano M, Rapino D, Comegna L, et al. Role of the C1858T polymorphism of protein tyrosine phosphatase non-receptor type 22 (*PTPN22*) in children and adolescents with type 1 diabetes. Pharmacogenomics J. 2017;17:186–191. 26902538 10.1038/tpj.2016.6

[43] Pawłowicz M, Filiów R, Krzykowski G, Stanisławska-Sachadyn A, Morzuch L, Kulczycka J, et al. Coincidence of *PTPN22* c.1858CC and *FCRL3* -169CC genotypes as a biomarker of preserved residual β-cell function in children with type 1 diabetes. Pediatr Diabetes. 2017;18:696–705. 27615679 10.1111/pedi.12429

