# Supplementary material for: PTPN22 intron polymorphism rs1310182 (c.2054-852T>C) is associated with type 1 diabetes mellitus in patients of Armenian descent
Source: PLoS One. 2023 Jun 14;18(6):e0286743. doi: 10.1371/journal.pone.0286743 (PMC10266637; doi:10.1371/journal.pone.0286743)

A - rs2476601

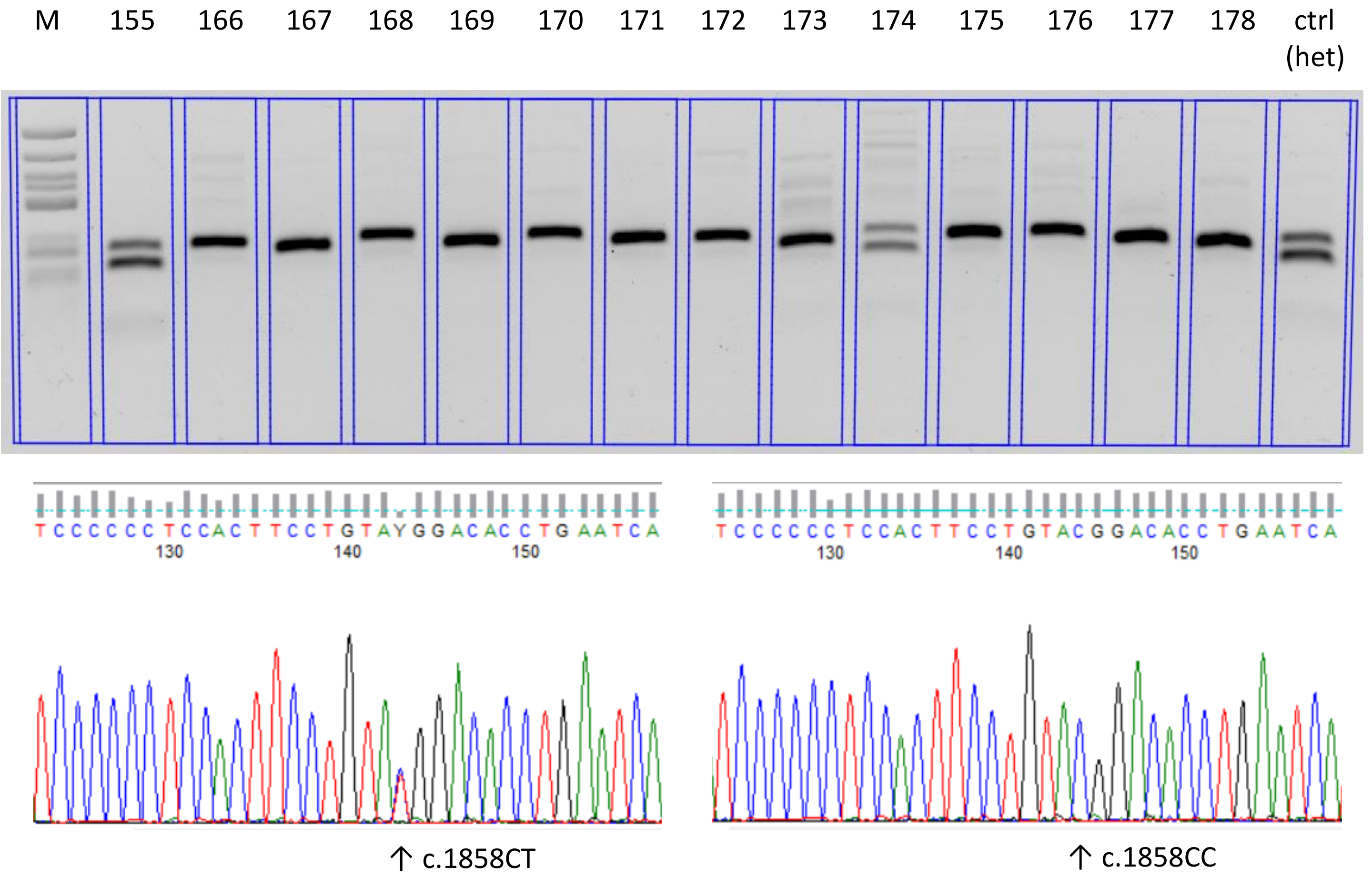

B - rs1310182

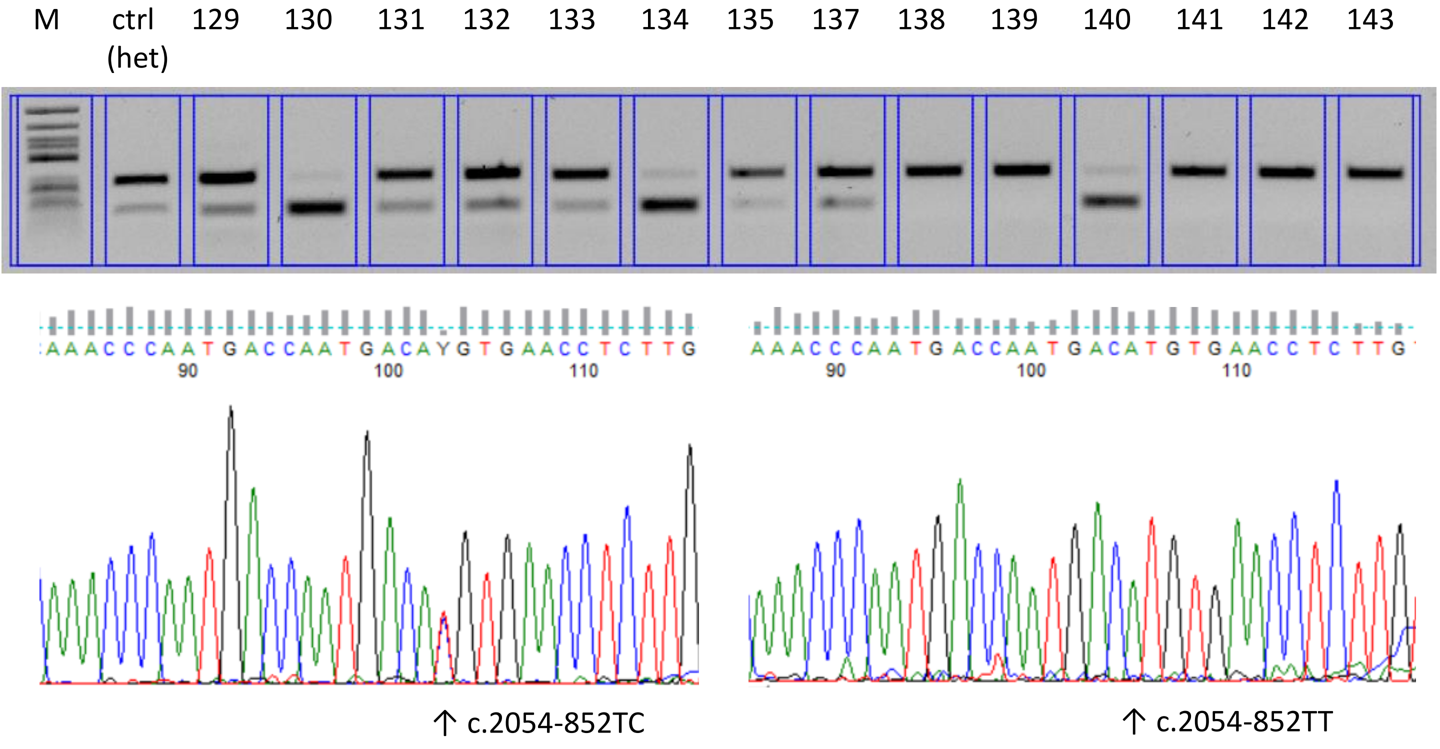

Supplement: S1 Fig — (A) rs2476601 and (B) rs1310182. Raw images are provided in S1 Raw images. (PDF) [file pone.0286743.s001.pdf]
