# Supplementary figures and images for: PTPN22 intron polymorphism rs1310182 (c.2054-852T>C) is associated with type 1 diabetes mellitus in patients of Armenian descent
Source: PLoS One. 2023 Jun 14;18(6):e0286743. doi: 10.1371/journal.pone.0286743 (PMC10266637; doi:10.1371/journal.pone.0286743)

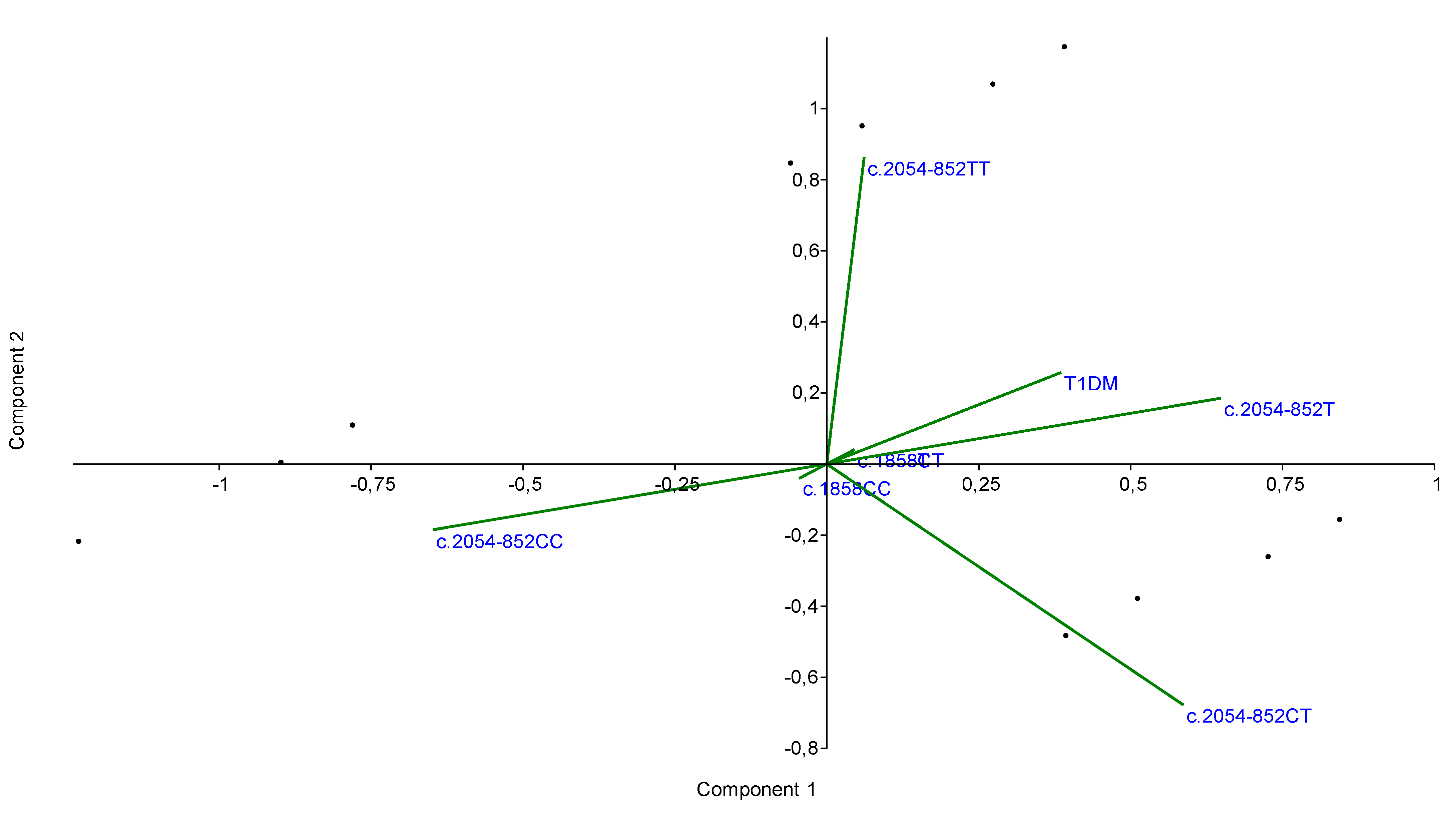

Supplement: S2 Fig — The biplot shows the outcomes of principal component analysis (PCA), which determined, whether any of the two genotyped PTPN22 SNPs (in sum six genotypes and two minor alleles) were associated with type 1 diabetes mellitus. The PCA simply reduced the data to only two variables that represent the two most important components (Axis 1 and Axis 2) and projected the correlation of the other variables with these components. The genotypes and minor alleles are indicated by the position of the polymorphism and relevant letters; T1DM = type 1 diabetes mellitus. Axis 1 explained 46.9% of the variability (eigenvalue 0.590) and Axis 2 explained 23.1% of the variability (eigenvalue 0.291). (TIF) [file pone.0286743.s002.tif]

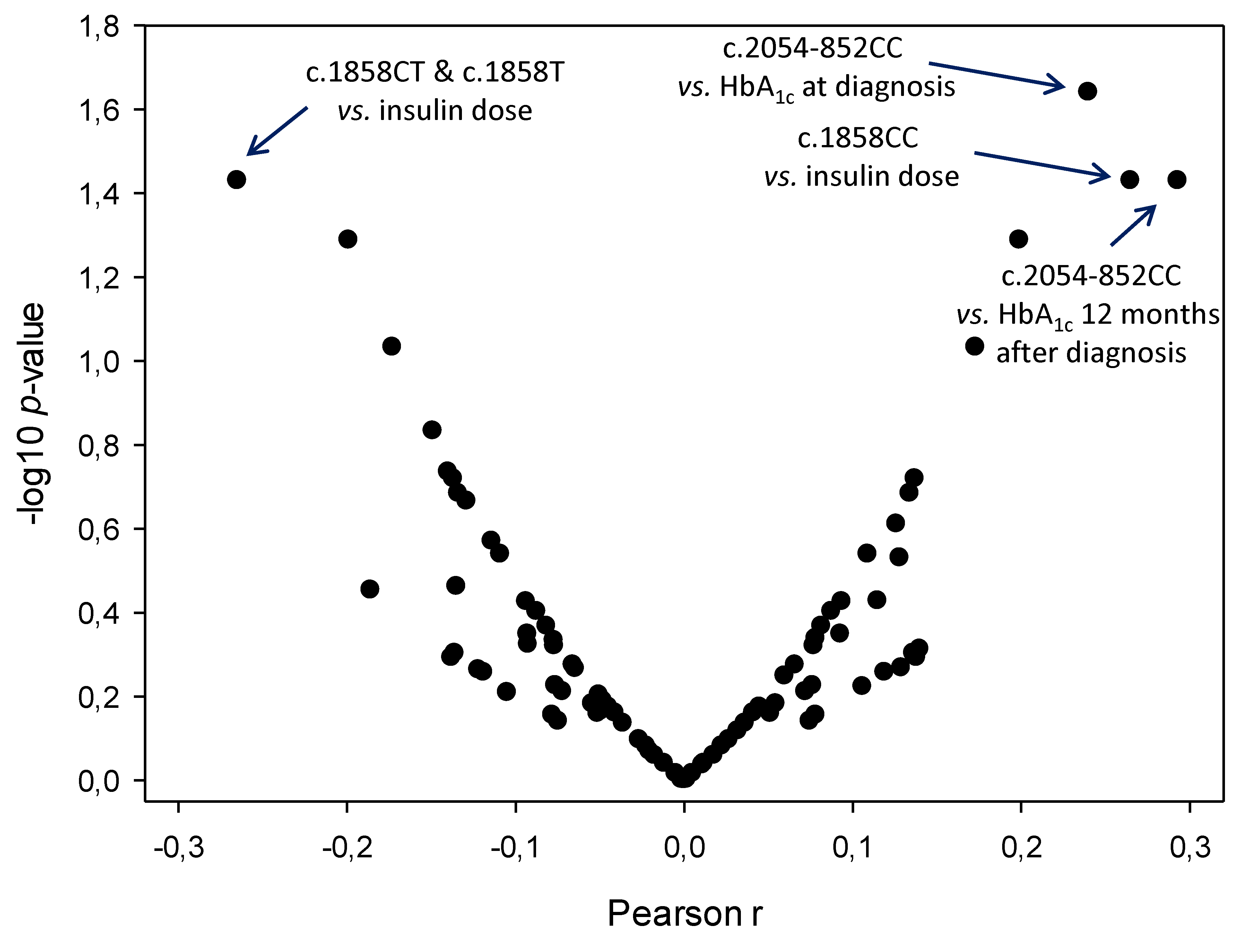

Supplement: S3 Fig — The data are shown as–log10-transformed p values and corresponding Pearson correlation coefficients r. The most significant values are associated with the descriptor of the respective data point. (TIF) [file pone.0286743.s003.tif]

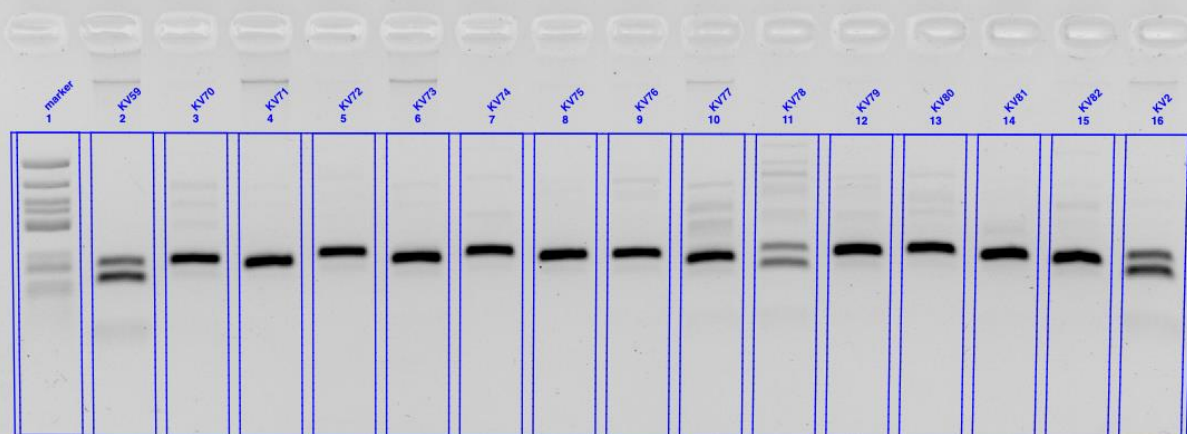

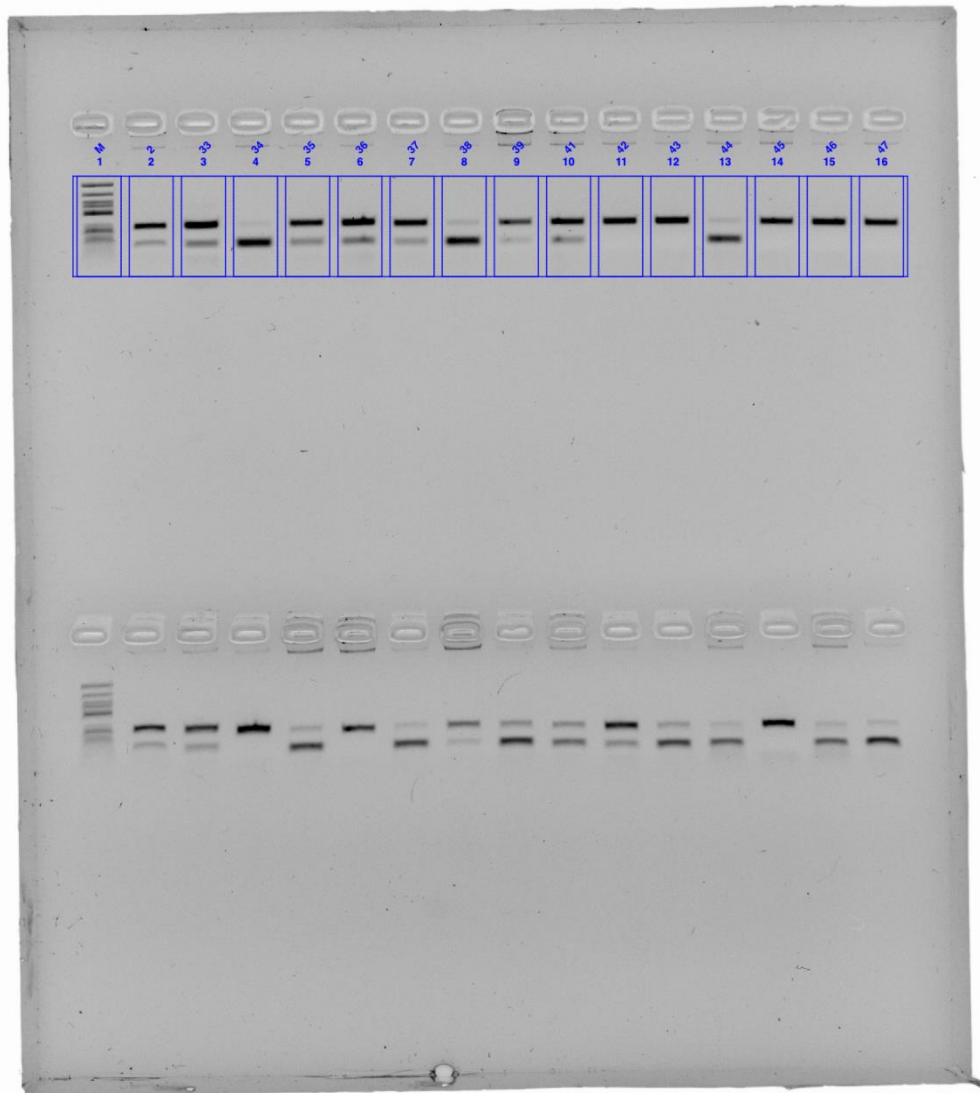

Raw gel figure of S1 Fig B

Supplement: S1 Raw images — (PDF) [file pone.0286743.s005.pdf]
